# Could the future of medical school examinations be open-book - a medical student’s perspective?

**DOI:** 10.1080/10872981.2020.1787308

**Published:** 2020-06-29

**Authors:** Georgia Mathieson, Roxanne Sutthakorn, Olivia Thomas

**Affiliations:** Faculty of Life Sciences and Medicine, King’s College London GKT School of Medical Education, London, UK

**Keywords:** Medical education, assessment, open-book examination, COVID-19

The stress and uncertainty of undertaking a medical degree and summative open-book examinations (OBE) amidst the COVID-19 pandemic is undeniable. However, we wanted to propose the question of a shift in medical written examinations becoming purely online OBEs in the future. We see this as a positive change for the future of medical education in contradiction to the negative stance taken by others around the open-book nature as well as negativity about continuing with medical education through a pandemic [1], something we see as crucial at this time.

Having completed our summative OBEs, overall, we believe the process was a fair assessment of our knowledge that was not drastically different due to the open-book nature, supported by analysis of data from Imperial College London [2]. Several steps were taken by our institution to overcome the anxieties and challenges highlighted by the authors. The unfamiliar format OBEs were tackled with a trial run of 25 questions using the assessment interface, the Medical Schools Council Assessment Alliance Assessment Suite (MSCAA). We found the MSCAA to be extremely user friendly with options to highlight and annotate questions, flag answers to come back to later and unanswered questions were clearly identifiable so that they were not missed. In comparison to traditional paper examinations, it was much easier to identify questions that had been flagged or left unanswered, as well as eliminating the transfer of answers to paper computer-marked answer sheets where errors commonly occur. Additionally, we were allocated 15 minutes extra per hour of the exam due to unfamiliarity using the MSCAA. Concerns about internet connection problems were mitigated as the MSCAA saved our answers automatically as we progressed through the examination; therefore, if the connection was lost, we could pick up where we left off. Furthermore, immediately after the examination, we were informed that if we had experienced any issues, we should file for mitigating circumstances with clear instructions on how to do this.

After completing our OBE, in hindsight, the use of Google and personal notes facilitated the answer to some of the simpler questions. However, these had mostly been committed to memory during revision and through clinical exposure due to their simple nature. Yet, more complicated problem-solving-based questions, often accompanied by investigation results or a long patient prologue, were much more difficult to answer using Google or notes. We appreciate that in clinical practice, resources are commonly used by the clinical team to aid treatment in the form of guidelines or referencing the British National Formulary (BNF) and we feel that OBEs are a fantastic way to learn to utilise these in a time-pressured environment. The purpose of progression through medical school is to ensure we are safe doctors, even in emergency situations guidelines are followed and advice over management is sought, safe junior doctors do not jump into treatment without consulting a senior if there is doubt. Overall, it is undeniable that students require a strong core knowledge, and this remained unchanged by the new OBEs. However, the style of our revision may have changed from memorising facts to learning how to utilise guidelines, but we feel this is a positive change that corresponds more closely to working life.

Despite the steps taken by the medical school, academic misconduct remained the main concern for our cohort. Students were most concerned about others ‘cheating’ rather than focusing on their own learning. We feel that it is easy to get side-tracked during exam season about learning purely for exams, and unfortunately, the competitive nature about exam results between medical students is reinforced by the ranking system that contributes towards the allocation of Foundation posts once qualified. However, we found comfort from remembering that we are learning to become safe doctors, well equipped with a good set of clinical knowledge rather than purely for exam grades. Regardless, cheating was addressed by the medical school and we were informed there would be strict formal disciplinary action for students found to be sharing answers or asking colleagues for help. Additionally, the randomised question order in a time-pressure examination meaning there simply was not time to be consulting with others.

We have recently been informed that all of our written examinations in our final year will take place as OBEs, and this is something we see as a positive change for the future. Overall, the OBE was not more anxiety-provoking, being able to complete it in a familiar and comfortable environment with simple home comforts. As medical education and the curriculum evolves, we envisage that OBEs could form more frequent and regular ‘knowledge checks’ as the logistical organisation is greatly reduced compared to traditional paper examinations. Undertaking more frequent OBEs also has the power to reduce overall anxiety around examinations [3].

Finally, we feel that the continued learning of medical students is vitally important and disagree with the view that ‘our time as medical students could be spent more productively’ [1]. The role of medical students, especially those entering their final year of study needs to be fully engaged in continued learning to ensure clinical competency to enter the National Health Service (NHS) as safe and effective junior doctors [4]. Although we recognise the ethical and moral dilemmas we face as medical students wanting to help out, ultimately to have the greatest impact we must be ready to join the NHS when it is our time. Cancelling examinations and teaching is not the answer to this. Continued learning in such uncertain times gives us structure to our days, which otherwise may be empty, gives us purpose and a goal to work towards, as well as a sense of accomplishment as we progress through.
